# 2-Bromo-2-methyl-*N*-*p*-tolyl­propanamide

**DOI:** 10.1107/S1600536811019337

**Published:** 2011-05-28

**Authors:** Rodolfo Moreno-Fuquen, David E. Quintero, Fabio Zuluaga, Alan R. Kennedy, Regina H. De Almeida Santos

**Affiliations:** a Departamento de Química, Facultad de Ciencias, Universidad del Valle, Apartado 25360, Santiago de Cali, Colombia; b WestCHEM, Department of Pure and Applied Chemistry, University of Strathclyde, 295 Cathedral Street, Glasgow G1 1XL, Scotland; c Instituto de Química de São Carlos, Universidade de São Paulo, USP, São Carlos, SP, Brazil

## Abstract

In the title mol­ecule, C_11_H_14_BrNO, there is twist between the mean plane of the amide group and the benzene ring [C(=O)—N—C C torsion angle = −31.2 (5)°]. In the crystal, inter­molecular N—H⋯O and weak C—H⋯O hydrogen bonds link mol­ecules into chains along [100]. The methyl group H atoms are disordered over two sets of sites with equal occupancy.

## Related literature

For initiators in ATRP processes (polymerization by atom transfer radical), see: Matyjaszewski & Xia (2001 ▶); Kato *et al.* (1995 ▶); Pietrasik & Tsarevsky (2010 ▶). For a related structure, see: Moreno-Fuquen *et al.* (2011 ▶). For hydrogen-bond graph sets, see: Etter (1990 ▶).
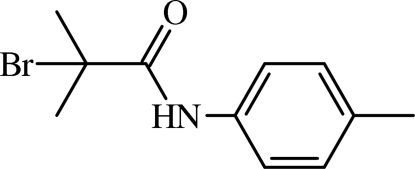


## Experimental

### 

#### Crystal data

C_11_H_14_BrNO
*M* *_r_* = 256.14
Orthorhombic, 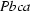

*a* = 10.0728 (4) Å
*b* = 11.2577 (4) Å
*c* = 20.3670 (6) Å
*V* = 2309.55 (14) Å^3^
*Z* = 8
Mo *K*α radiation
μ = 3.53 mm^−1^
*T* = 123 K
0.25 × 0.12 × 0.05 mm

#### Data collection

Oxford Diffraction Xcalibur E diffractometer
Absorption correction: multi-scan (*CrysAlis PRO*; Oxford Diffraction, 2009 ▶) *T*_min_ = 0.751, *T*_max_ = 1.000
10140 measured reflections
2762 independent reflections
1819 reflections with *I* > 2σ(*I*)
*R*_int_ = 0.049

#### Refinement

*R*[*F* ^2^ > 2σ(*F* ^2^)] = 0.045
*wR*(*F* ^2^) = 0.093
*S* = 1.06
2762 reflections
133 parameters
H atoms treated by a mixture of independent and constrained refinement
Δρ_max_ = 0.58 e Å^−3^
Δρ_min_ = −0.46 e Å^−3^

### 

Data collection: *CrysAlis CCD* (Oxford Diffraction, 2009 ▶); cell refinement: *CrysAlis CCD*; data reduction: *CrysAlis CCD*; program(s) used to solve structure: *SHELXS97* (Sheldrick, 2008 ▶); program(s) used to refine structure: *SHELXL97* (Sheldrick, 2008 ▶); molecular graphics: *ORTEP-3 for Windows* (Farrugia, 1997 ▶) and *Mercury* (Macrae *et al.*, 2006 ▶); software used to prepare material for publication: *WinGX* (Farrugia, 1999 ▶) and *PARST* (Nardelli, 1995 ▶).

## Supplementary Material

Crystal structure: contains datablocks I, global. DOI: 10.1107/S1600536811019337/lh5254sup1.cif

Structure factors: contains datablocks I. DOI: 10.1107/S1600536811019337/lh5254Isup2.hkl

Supplementary material file. DOI: 10.1107/S1600536811019337/lh5254Isup3.cml

Additional supplementary materials:  crystallographic information; 3D view; checkCIF report

## Figures and Tables

**Table 1:** Hydrogen-bond geometry (Å, °)

*D*—H⋯*A*	*D*—H	H⋯*A*	*D*⋯*A*	*D*—H⋯*A*
N1—H1*N*⋯O1^i^	0.84 (2)	2.13 (2)	2.937 (3)	161 (3)
C1—H1*C*⋯O1^i^	0.98	2.44	3.382 (4)	162
C10—H10⋯O1^i^	0.95	2.57	3.321 (4)	137

Symmetry code: (i) 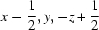.

**Fig. 1.**
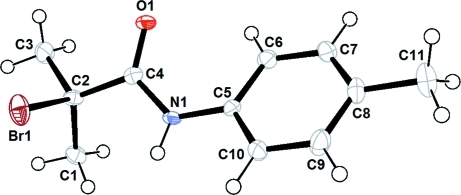
An ORTEP-3 (Farrugia, 1997) plot of (I) with displacement ellipsoids drawn at the 50% probability level. H atoms are shown as spheres of arbitary radius.

**Fig. 2.**
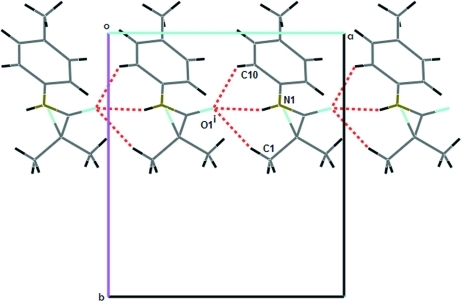
Part of the crystal structure of (I), showing the formation of a one dimensional chain along [100]. Symmetry code: (i) x-1/2,+y,-z+1/2.

## supplementary crystallographic information

### Comment

The ATRP process (polymerization by atom transfer radical) allows control of the composition and functionality in polymerization reactions (Pietrasik & Tsarevsky, 2010). The use of functional initiators in these reactions allows the synthesis of new materials. Most initiators for ATRP processes are alkyl halides (Matyjaszewski & Xia, 2001; Kato *et al.*, 1995). As part of our work related to functional initiators in polymerization processes (Moreno-Fuquen *et al.*, 2011) we have determined the crystal structure of the title compound (I). The molecular structure of (I) is shown in Fig. 1. There is a twist between the mean plane of the amide group and benzene ring giving a C4—N1—C5—C6 torsion angle of -31.2 (5) °. In the crystal, intermolecular N—H···O and weak C—H···O hydrogen bonds link molecules into one-dimensional chains along [100] incorporating C(4) graph motifs (Etter, 1990) (see Table 1 and Fig. 2).

### Experimental

The initial reagents were purchased from Aldrich Chemical Co. and were used as received. In a 100mL round bottom flask 4-methylaniline (3.173 mmoles, 0.340 g), triethylamine (0.635 mmol, 0.064 g) were mixed, then a solution of 2-bromo isobutiryl bromide (0.685 g) in anhydrous THF (5 ml) was added drop wise, under an argon stream. The reaction was carried out in a dry bag overnight under magnetic stirring. The solid was filtered off and dichloromethane (20 ml) added to the organic phase which was washed with brine (50 ml) followed by water (10 ml). The solution was concentrated at low pressure affording colourless crystals and recrystalized from a solution of hexane and ethyl acetate (80:20). M.p. 364 (1) K.

### Refinement

The H-atoms were placed geometrically  with C—H= 0.95 Å for aromatic, C—H = 0.98 Å for methyl and  U_iso_(H) 1.2 and 1.5 times U_eq_ of the parent atom respectively.  The methyl group H atoms are disordered over two sets of sites with equal occupancy.

### Crystal data

**Table:**

C_11_H_14_BrNO	*D*_x_ = 1.473 Mg m^−^^3^
*M**_r_* = 256.14	Melting point: 385(1) K
Orthorhombic, *P**b**c**a*	Mo *K*α radiation, λ = 0.71073 Å
Hall symbol: -P 2ac 2ab	Cell parameters from 2266 reflections
*a* = 10.0728 (4) Å	θ = 3.4–29.5°
*b* = 11.2577 (4) Å	µ = 3.53 mm^−^^1^
*c* = 20.3670 (6) Å	*T* = 123 K
*V* = 2309.55 (14) Å^3^	Tablet, colourless
*Z* = 8	0.25 × 0.12 × 0.05 mm
*F*(000) = 1040	

### Data collection

**Table:**

Oxford Diffraction Xcalibur E diffractometer	2762 independent reflections
Radiation source: fine-focus sealed tube	1819 reflections with *I* > 2σ(*I*)
graphite	*R*_int_ = 0.049
ω scans	θ_max_ = 28.0°, θ_min_ = 3.4°
Absorption correction: multi-scan (*CrysAlis PRO*; Oxford Diffraction, 2009)	*h* = −12→13
*T*_min_ = 0.751, *T*_max_ = 1.000	*k* = −10→14
10140 measured reflections	*l* = −26→22

### Refinement

**Table:**

Refinement on *F*^2^	Primary atom site location: structure-invariant direct methods
Least-squares matrix: full	Secondary atom site location: difference Fourier map
*R*[*F*^2^ > 2σ(*F*^2^)] = 0.045	Hydrogen site location: inferred from neighbouring sites
*wR*(*F*^2^) = 0.093	H atoms treated by a mixture of independent and constrained refinement
*S* = 1.06	*w* = 1/[σ^2^(*F*_o_^2^) + (0.0316*P*)^2^ + 0.7321*P*] where *P* = (*F*_o_^2^ + 2*F*_c_^2^)/3
2762 reflections	(Δ/σ)_max_ < 0.001
133 parameters	Δρ_max_ = 0.58 e Å^−^^3^
0 restraints	Δρ_min_ = −0.46 e Å^−^^3^

### Special details

**Table:**

Geometry. All esds (except the esd in the dihedral angle between two l.s. planes) are estimated using the full covariance matrix. The cell esds are taken into account individually in the estimation of esds in distances, angles and torsion angles; correlations between esds in cell parameters are only used when they are defined by crystal symmetry. An approximate (isotropic) treatment of cell esds is used for estimating esds involving l.s. planes.
Refinement. Refinement of F^2^ against ALL reflections. The weighted R-factor wR and goodness of fit S are based on F^2^, conventional R-factors R are based on F, with F set to zero for negative F^2^. The threshold expression of F^2^ > 2sigma(F^2^) is used only for calculating R-factors(gt) etc. and is not relevant to the choice of reflections for refinement. R-factors based on F^2^ are statistically about twice as large as those based on F, and R- factors based on ALL data will be even larger.

### Fractional atomic coordinates and isotropic or equivalent isotropic displacement parameters (Å^2^)

**Table:**

	*x*	*y*	*z*	*U*_iso_*/*U*_eq_	Occ. (<1)
Br1	0.74332 (3)	0.28652 (3)	0.384590 (15)	0.02982 (13)	
O1	0.94616 (17)	0.2851 (2)	0.24129 (10)	0.0200 (5)	
N1	0.7262 (2)	0.2730 (3)	0.21911 (12)	0.0158 (6)	
C1	0.6864 (3)	0.4811 (3)	0.30022 (16)	0.0214 (7)	
H1A	0.7056	0.5288	0.2611	0.032*	
H1B	0.6768	0.5335	0.3384	0.032*	
H1C	0.6037	0.4368	0.2935	0.032*	
C2	0.7996 (3)	0.3946 (3)	0.31211 (15)	0.0173 (7)	
C3	0.9232 (3)	0.4587 (3)	0.33541 (16)	0.0287 (9)	
H3A	0.9914	0.4004	0.3472	0.043*	
H3B	0.9015	0.5073	0.3739	0.043*	
H3C	0.9565	0.5100	0.3002	0.043*	
C4	0.8312 (3)	0.3116 (3)	0.25448 (14)	0.0158 (7)	
C5	0.7307 (2)	0.1928 (3)	0.16557 (14)	0.0160 (7)	
C6	0.8384 (3)	0.1847 (3)	0.12261 (14)	0.0203 (7)	
H6	0.9135	0.2348	0.1281	0.024*	
C7	0.8347 (3)	0.1030 (3)	0.07213 (15)	0.0241 (8)	
H7	0.9082	0.0986	0.0431	0.029*	
C8	0.7285 (3)	0.0274 (3)	0.06198 (15)	0.0229 (8)	
C9	0.6210 (3)	0.0383 (3)	0.10438 (16)	0.0273 (8)	
H9	0.5457	−0.0113	0.0985	0.033*	
C10	0.6216 (3)	0.1202 (3)	0.15499 (15)	0.0225 (8)	
H10	0.5463	0.1267	0.1829	0.027*	
C11	0.7287 (3)	−0.0636 (4)	0.00758 (17)	0.0338 (9)	
H11A	0.6452	−0.1082	0.0085	0.051*	0.50
H11B	0.8033	−0.1184	0.0138	0.051*	0.50
H11C	0.7378	−0.0233	−0.0348	0.051*	0.50
H11D	0.8123	−0.0584	−0.0168	0.051*	0.50
H11E	0.6542	−0.0482	−0.0222	0.051*	0.50
H11F	0.7197	−0.1433	0.0264	0.051*	0.50
H1N	0.652 (2)	0.292 (3)	0.2342 (15)	0.032 (10)*	

### Atomic displacement parameters (Å^2^)

**Table:**

	*U*^11^	*U*^22^	*U*^33^	*U*^12^	*U*^13^	*U*^23^
Br1	0.0475 (2)	0.0235 (2)	0.01844 (18)	0.00201 (17)	0.00410 (15)	0.00302 (15)
O1	0.0101 (9)	0.0260 (14)	0.0240 (12)	0.0013 (10)	−0.0012 (8)	−0.0053 (11)
N1	0.0086 (12)	0.0198 (15)	0.0191 (13)	0.0006 (11)	0.0013 (9)	−0.0026 (11)
C1	0.0213 (16)	0.017 (2)	0.0254 (18)	0.0021 (13)	0.0028 (14)	−0.0020 (15)
C2	0.0131 (13)	0.0159 (19)	0.0228 (17)	−0.0020 (12)	0.0012 (12)	0.0001 (15)
C3	0.0218 (16)	0.037 (3)	0.0269 (19)	−0.0069 (16)	−0.0011 (14)	−0.0152 (18)
C4	0.0163 (14)	0.0137 (18)	0.0175 (15)	−0.0003 (12)	−0.0010 (12)	0.0051 (14)
C5	0.0137 (14)	0.0189 (18)	0.0153 (15)	0.0014 (13)	−0.0025 (11)	0.0010 (13)
C6	0.0172 (14)	0.025 (2)	0.0182 (16)	−0.0014 (13)	0.0012 (12)	0.0021 (15)
C7	0.0217 (16)	0.034 (2)	0.0165 (17)	0.0033 (15)	0.0025 (13)	−0.0009 (16)
C8	0.0334 (19)	0.020 (2)	0.0155 (16)	0.0022 (15)	−0.0021 (14)	−0.0022 (14)
C9	0.0275 (17)	0.031 (2)	0.0230 (18)	−0.0104 (15)	−0.0014 (14)	−0.0048 (16)
C10	0.0182 (15)	0.030 (2)	0.0192 (17)	−0.0032 (14)	0.0029 (13)	−0.0042 (16)
C11	0.051 (2)	0.029 (2)	0.0213 (18)	−0.0049 (17)	0.0051 (16)	−0.0064 (16)

### Geometric parameters (Å, °)

**Table:**

Br1—C2	1.995 (3)	C6—C7	1.380 (4)
O1—C4	1.226 (3)	C6—H6	0.9500
N1—C4	1.352 (4)	C7—C8	1.382 (4)
N1—C5	1.416 (4)	C7—H7	0.9500
N1—H1N	0.840 (17)	C8—C9	1.391 (4)
C1—C2	1.519 (4)	C8—C11	1.509 (5)
C1—H1A	0.9800	C9—C10	1.382 (4)
C1—H1B	0.9800	C9—H9	0.9500
C1—H1C	0.9800	C10—H10	0.9500
C2—C3	1.515 (4)	C11—H11A	0.9800
C2—C4	1.533 (4)	C11—H11B	0.9800
C3—H3A	0.9800	C11—H11C	0.9800
C3—H3B	0.9800	C11—H11D	0.9800
C3—H3C	0.9800	C11—H11E	0.9800
C5—C10	1.387 (4)	C11—H11F	0.9800
C5—C6	1.397 (4)		
			
C4—N1—C5	126.2 (2)	C8—C7—H7	118.5
C4—N1—H1N	115 (2)	C7—C8—C9	117.1 (3)
C5—N1—H1N	118 (2)	C7—C8—C11	121.8 (3)
C2—C1—H1A	109.5	C9—C8—C11	121.1 (3)
C2—C1—H1B	109.5	C10—C9—C8	121.2 (3)
H1A—C1—H1B	109.5	C10—C9—H9	119.4
C2—C1—H1C	109.5	C8—C9—H9	119.4
H1A—C1—H1C	109.5	C9—C10—C5	120.8 (3)
H1B—C1—H1C	109.5	C9—C10—H10	119.6
C3—C2—C1	111.2 (3)	C5—C10—H10	119.6
C3—C2—C4	111.1 (2)	C8—C11—H11A	109.5
C1—C2—C4	115.1 (2)	C8—C11—H11B	109.5
C3—C2—Br1	107.0 (2)	H11A—C11—H11B	109.5
C1—C2—Br1	107.20 (19)	C8—C11—H11C	109.5
C4—C2—Br1	104.7 (2)	H11A—C11—H11C	109.5
C2—C3—H3A	109.5	H11B—C11—H11C	109.5
C2—C3—H3B	109.5	C8—C11—H11D	109.5
H3A—C3—H3B	109.5	H11A—C11—H11D	141.1
C2—C3—H3C	109.5	H11B—C11—H11D	56.3
H3A—C3—H3C	109.5	H11C—C11—H11D	56.3
H3B—C3—H3C	109.5	C8—C11—H11E	109.5
O1—C4—N1	123.0 (3)	H11A—C11—H11E	56.3
O1—C4—C2	120.8 (3)	H11B—C11—H11E	141.1
N1—C4—C2	116.2 (2)	H11C—C11—H11E	56.3
C10—C5—C6	118.7 (3)	H11D—C11—H11E	109.5
C10—C5—N1	118.1 (3)	C8—C11—H11F	109.5
C6—C5—N1	123.3 (3)	H11A—C11—H11F	56.3
C7—C6—C5	119.3 (3)	H11B—C11—H11F	56.3
C7—C6—H6	120.4	H11C—C11—H11F	141.1
C5—C6—H6	120.4	H11D—C11—H11F	109.5
C6—C7—C8	122.9 (3)	H11E—C11—H11F	109.5
C6—C7—H7	118.5		
			
C5—N1—C4—O1	4.0 (5)	C10—C5—C6—C7	−1.6 (5)
C5—N1—C4—C2	−177.5 (3)	N1—C5—C6—C7	179.3 (3)
C3—C2—C4—O1	14.6 (4)	C5—C6—C7—C8	−0.4 (5)
C1—C2—C4—O1	142.0 (3)	C6—C7—C8—C9	1.6 (5)
Br1—C2—C4—O1	−100.5 (3)	C6—C7—C8—C11	−178.2 (3)
C3—C2—C4—N1	−163.9 (3)	C7—C8—C9—C10	−1.0 (5)
C1—C2—C4—N1	−36.5 (4)	C11—C8—C9—C10	178.8 (3)
Br1—C2—C4—N1	81.0 (3)	C8—C9—C10—C5	−0.9 (5)
C4—N1—C5—C10	149.7 (3)	C6—C5—C10—C9	2.2 (5)
C4—N1—C5—C6	−31.2 (5)	N1—C5—C10—C9	−178.6 (3)

### Hydrogen-bond geometry (Å, °)

**Table:**

*D*—H···*A*	*D*—H	H···*A*	*D*···*A*	*D*—H···*A*
N1—H1N···O1^i^	0.84 (2)	2.13 (2)	2.937 (3)	161 (3)
C1—H1C···O1^i^	0.98	2.44	3.382 (4)	162
C10—H10···O1^i^	0.95	2.57	3.321 (4)	137

Symmetry codes: (i) *x*−1/2, *y*, −*z*+1/2.
